# Correction: Pettenuzzo et al. Mechanical Behaviour of Plantar Adipose Tissue: From Experimental Tests to Constitutive Analysis. *Bioengineering* 2024, *11*, 42

**DOI:** 10.3390/bioengineering12030226

**Published:** 2025-02-24

**Authors:** Sofia Pettenuzzo, Elisa Belluzzi, Assunta Pozzuoli, Veronica Macchi, Andrea Porzionato, Rafael Boscolo-Berto, Pietro Ruggieri, Alice Berardo, Emanuele Luigi Carniel, Chiara Giulia Fontanella

**Affiliations:** 1 Department of Civil, Environmental and Architectural Engineering, University of Padova, 35131 Padova, Italy; sofia.pettenuzzo@unipd.it (S.P.); alice.berardo@unipd.it (A.B.); 2 Musculoskeletal Pathology and Oncology Laboratory, Department of Surgery, Oncology and Gastroenterology, University of Padova (DiSCOG), Via Giustiniani 3, 35128 Padova, Italy; elisa.belluzzi@unipd.it (E.B.); assunta.pozzuoli@unipd.it (A.P.); 3 Orthopedics and Orthopedic Oncology, Department of Surgery, Oncology and Gastroenterology (DiSCOG), University of Padova, Via Giustiniani 3, 35128 Padova, Italy; pietro.ruggieri@unipd.it; 4 Centre for Mechanics of Biological Materials, University of Padova, 35131 Padova, Italy; veronica.macchi@unipd.it (V.M.); andrea.porzionato@unipd.it (A.P.); rafael.boscoloberto@unipd.it (R.B.-B.); emanueleluigi.carniel@unipd.it (E.L.C.); 5 Department of Neuroscience, Institute of Human Anatomy, University of Padova, 35121 Padova, Italy; 6 Veneto Region Reference Center for the Preservation and Use of Gifted Bodies, Veneto Region, 35121 Padua, Italy; 7 Department of Biomedical Sciences, University of Padova, 35131 Padova, Italy; 8 Department of Industrial Engineering, University of Padova, 35131 Padova, Italy

## Affiliation Correction

In the original publication [1], the postcode of Affiliation 6 should be 35121.

## Error in Figure

In the original publication [1], there was a mistake in Figure 4 as published.

The modification to Figure 4 only concerns the part that is marked with the letter a. In Section 2.2.1. (Unconfined Compression Tests), we explain that with the stress relaxation procedure, the sample reached a 60% compression strain at the end of the testing approach, but as reported in Section 3 (Results), we decided to limit the strain up to 50%. Therefore, we took the stress–strain curve which considers the strain in the range from 0 to 0.6 and, with software for curve elaboration, stopped the deformation up to 0.5 (50%). Unfortunately, while performing this procedure, we did not update the values along the *y*-axis, which remain those corresponding to the 0–0.6 range, 70 kPa, instead of the correct 14 kPa. The updated visual representation is provided below. Hence, Figure 4 should be updated to have the correct *y*-axis range of values 0–14 kPa, in part a.

The corrected Figure 4 appears below. The authors state that the scientific conclusions are unaffected. This correction was approved by the Academic Editor. The original publication has also been updated.

**Figure 4:**
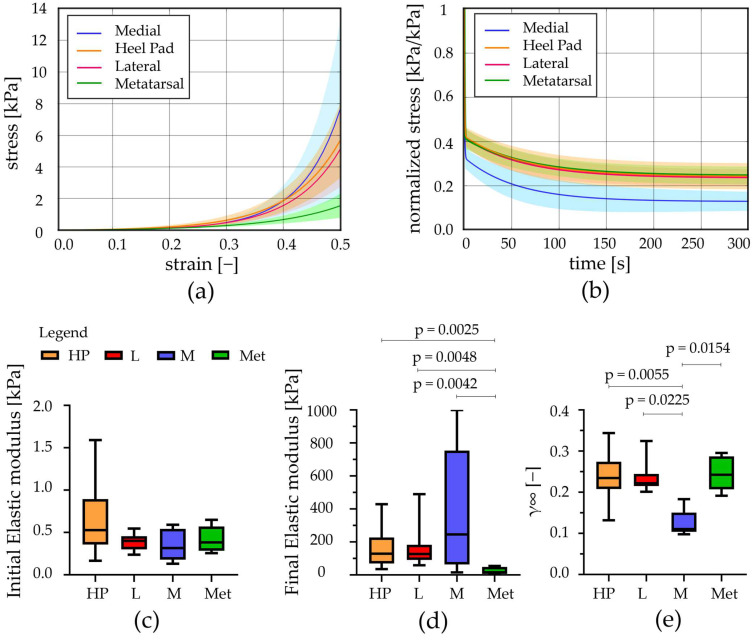
Unconfined compression tests: (**a**) Stress–strain equilibrium curves and (**b**) normalized stress–time results comparing the different regions (mean ± SD). (**c**) Initial and (**d**) final elastic modulus computed from stress–strain equilibrium curves, (**e**) relative stiffness computed at t = 300 s (median with maximum and minimum values). HP = heel pad; L = lateral; M = medial; Met = metatarsal; *p* = statistically different *p*-value.
